# A multi-parameterized artificial neural network for lung cancer risk prediction

**DOI:** 10.1371/journal.pone.0205264

**Published:** 2018-10-24

**Authors:** Gregory R. Hart, David A. Roffman, Roy Decker, Jun Deng

**Affiliations:** Department of Therapeutic Radiology, School of Medicine, Yale University, New Haven, Connecticut, United States of America; UNITED STATES

## Abstract

The objective of this study is to train and validate a multi-parameterized artificial neural network (ANN) based on personal health information to predict lung cancer risk with high sensitivity and specificity. The 1997-2015 National Health Interview Survey adult data was used to train and validate our ANN, with inputs: gender, age, BMI, diabetes, smoking status, emphysema, asthma, race, Hispanic ethnicity, hypertension, heart diseases, vigorous exercise habits, and history of stroke. We identified 648 cancer and 488,418 non-cancer cases. For the training set the sensitivity was 79.8% (95% CI, 75.9%-83.6%), specificity was 79.9% (79.8%-80.1%), and AUC was 0.86 (0.85-0.88). For the validation set sensitivity was 75.3% (68.9%-81.6%), specificity was 80.6% (80.3%-80.8%), and AUC was 0.86 (0.84-0.89). Our results indicate that the use of an ANN based on personal health information gives high specificity and modest sensitivity for lung cancer detection, offering a cost-effective and non-invasive clinical tool for risk stratification.

## Introduction

Approximately 14% of new cancer cases each year in the United States are lung cancer, but the number of deaths related to lung cancer exceed those from breast, prostate, and colon cancers combined [1]. Even though it is well documented that smoking is the main causal factor, a predictive model that incorporates the synergetic effects of a multitude of patient-related factors and other health information would be useful in the evaluation of persons perceived to be a risk. In this work, we assess the aptitude of such a model developed from training an artificial neural network with the National Health Interview Survey (NHIS) datasets.

There are three main types of lung cancer: non-small cell lung cancer (about 85%), small cell lung cancer (about 10-15%), and lung carcinoid tumors (fewer than 5%) [2]. The standard method of detection by screening is low-dose computed tomography (LDCT) [3]. However, the Centers for Disease Control and Prevention (CDC) indicate that repeated exposure to low dose radiation increases cancer risk. The United States Preventive Services Task Force (USPSTF) recommends screening only for those who have 30 pack years or more of smoking and are current smokers or have stopped within the last 15 years, and are 55-80 years old [4]. While smoking is the primary risk factor, there are other relevant factors such as a family history of cancer, diet, and exposure to environmental tobacco smoke (second-hand smoke), radon, asbestos, or other carcinogens [5]. Lung cancers detected at a local stage has a 55% 5-year survival rate; however the majority of lung cancer patients are diagnosed with more advanced disease, with much lower survival rates (overall 5-year survival rate of 18%) [6].

The high risk population identified by the USPSTF lung cancer screening criteria is estimated to include approximately 8—9 million individuals in the United States. While the USPSTF recommendation represents an enormous step forward in early detection for lung cancer, there is ongoing debate as to whether the criteria include individuals whose risk is not high enough to warrant screening [7] and conversely exclude other individuals whose risk is demonstrably high by modeling studies [8, 9]. There has been intense interest in developing methods to more accurately identify individuals at high risk for lung cancer that incorporate demographic as well as biologic inputs. Accordingly many models have been created using a variety of methods such as logistic regression [10–12], restricted cubic splines [8, 13], and two-stage clonal expansion models [14, 15]. These methods have had varying success with AUCs of 0.57-0.88 with the average a little over 0.7 [16]. To the best of our knowledge, our work is the first study that uses machine learning algorithms on this type of data to predict lung cancer risk.

The aim of this study is to investigate a novel approach in predicting lung cancer risk, using a multi-parameterized artificial neural network (ANN) based on personal health information extracted from the National Health Interview Survey (NHIS) datasets. We hypothesized that a multi-parameterized ANN model using readily available clinical and demographic information commonly found in the electronic medical record (EMR) systems would be an effective clinical tool to predict and stratify lung cancer risk for individuals.

## Materials and methods

### Datasets and patient selection

We obtained NHIS adult survey files, from the CDC website, related to clinical and demographic status, including the corresponding manuals and criteria, which vary by year [17]. We used the NHIS survey datasets from 1997-2015, with the exception of 2004 due to known inaccuracies in the data file. The response rate for the NHIS adult survey is about 80% and we can only view the data that has been collected and filtered by the NHIS [18].

The USPSTF criteria for lung cancer screening guidelines are well defined. However, there is ongoing discussion regarding groups at high risk that are identifiable by modeling but are currently excluded from screening as they do not fit the USPSTF guideline [8, 9]. We decided to include the entire NHIS adult population on the basis of including as many cases of lung cancer as possible. We selected model inputs based on known or putative lung cancer risks factors, as well as clinical and demographic information in the dataset: age [1], body mass index (BMI) [19], diabetic status [20], smoking status, emphysema [21], asthma [22], race [23], Hispanic ethnicity [23], hypertension [24], heart diseases, vigorous exercise habits [25], and history of stroke [26]. The demographics of the entire sample used are shown in Table 1.

**Table 1:** The demographics of the NHIS dataset that was used in our ANN. We show means and standard deviations for the continuous variables, means for the binary variables, and the percentage for each race.

Input	Lung Cancer	Non-Cancer
Age	65.6 (±11.8)	46.1 (±17.6)
BMI	25.8 (±5.9)	27.3 (±6.0)
Heart Disease Score	0.13 (±0.22)	0.040 (±0.13)
Number of Vigorous Exercise done per week	0.38 (±1.7)	1.60 (3.0)
Female	53.8%	54.9%
Ever Smoked	83.8%	41.8%
Has Emphysema	24.1%	1.53%
Has Asthma	18.8%	11.2%
Has Diabetes	17.4%	7.92%
Ever Had a Stroke	9.57%	2.55%
Has Hypertension	18.8%	11.2%
Hispanic Ethnicity	7.10%	16.7%
Race:		
Caucasian	82.4%	77.3%
African American	14.2%	15.3%
Asian	1.39%	4.96%
Native American/Alaska Native	0.309%	0.868%
Multiracial	1.70%	1.55%

We used 70% of the data (454 lung cancer cases and 341,893 never cancer cases) for training and 30% for validation (195 lung cancer cases and 146,524 never cancer cases) with the selection being randomized for each group. Lung cancer cases meeting the inclusion criteria were limited to patients with lung cancer as the first diagnosed malignancy that occurred within 4 years of the survey date. Several of the inputs for our ANN are time-dependent, such as BMI and diabetic status. We selected a four-year cutoff as a compromise between the time-dependent aspects of the problem and the sample size restriction required for training and validation. Note that this four-year cutoff only applies to the lung cancer cases. The prevalence of cancer in our training and testing set is about twice the annual incidence rate [6]

### A multi-parameterized artificial neural network (ANN)

Andoni *et al* have explored the theoretical limits of neural networks with two hidden layers and have shown their ability to represent polynomial functions [27]. Thus we hypothesized that a two-layered neural network with a sufficient number of inputs and neurons would be able to make accurate cancer risk.

Based on success with non-melanoma skin cancer [28] we used 12 neurons per hidden layer. However we also explored networks with various numbers of neurons (6, 10, 11, 13, and 20) in each layer, none of which preformed significantly better.

**Fig 1:**
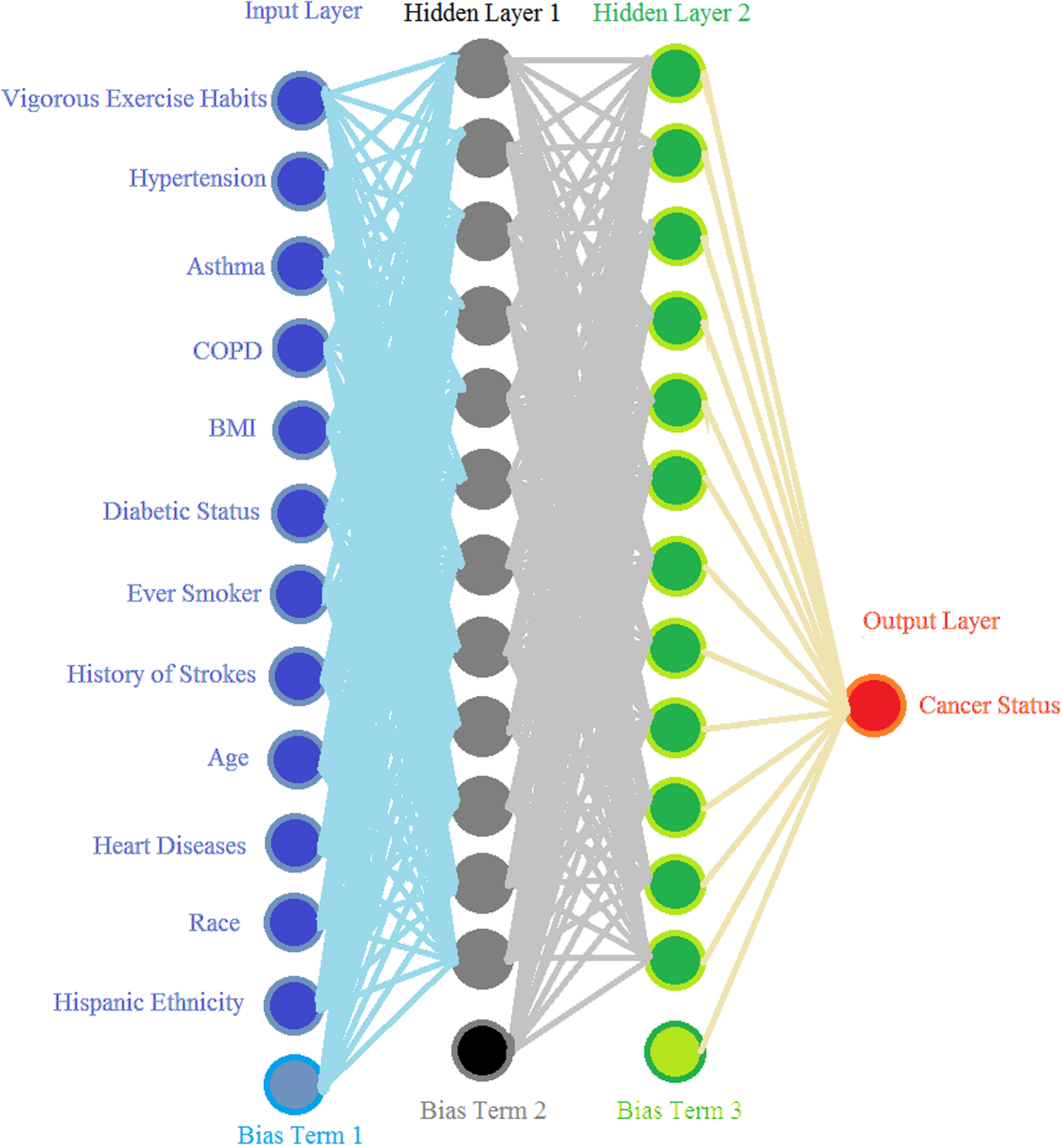
A sketch of our ANN. All lines are weights connecting one layer to next, with each circle either being an input, neuron, or output. The bias terms are analogous to intercepts and they improve the model’s performance.

A schematic of our ANN is shown in Fig 1. Our ANN uses a backpropagation algorithm with bias terms and gradient descent (simultaneously using all examples in the training dataset each epoch) [29]. Inputs were normalized to fall in between 0 and 1 with sigmoidal activation functions being used throughout. A modification was made to allow further speedup of convergence by increasing the learning rate 1% each time the cost function decreases and decreasing the learning rate 5% while resetting the weights to the last iteration if the cost function increases, similar to the momentum approach [30]. The network weights were randomly initialized between -1 and 1, biases were initialized to 1, and the learning rate started at 10.

Listed in Table 2 are all the personal health inputs to our ANN. Some inputs were rescaled to comply with the mathematical format required in ANN while others take binary inputs.

**Table 2:** A description of the inputs used in our ANN.

Input	Input Type	Input Range	Details
Age	Continuous	0-1	18-85, (85+ recorded as 85)
BMI	Continuous	0-1	BMI of 99.95+ recorded as 99.95
Heart Disease Score	Continuous	0-1	Coronary heart disease, Angina, Heart attacks, and other heart complications each contribute 0.25 to the score
Vigorous Exercise	Continuous	0-1	Number of times per week vigorous exercise is performed; 28+ is treated as 28. Minimum time for exercise to count was 10 minutes, except for the first half of 1997 for which it was 20 minutes.
Gender	Binary	0 or 1	0 is a man and 1 is a woman
Ever Smoked	Binary	0 or 1	Never smoked is 0 and current and former smokers are 1
Emphysema	Binary	0 or 1	No COPD is 0 and COPD is 1
Asthma	Binary	0 or 1	No asthma is 0 and asthma is 1
Diabetes	Binary	0 or 1	Non-diabetics and pre-diabetics are 0, with diabetics being 1
Strokes	Binary	0 or 1	No stroke is 0 and a prior stroke is 1
Hypertension	Binary	0 or 1	No hypertension is 0, and having single measurement of it is 1
Hispanic Ethnicity	Binary	0 or 1	Non-Hispanic is 0 and Hispanic is 1
Race	Continuous	0-1	Each race is assigned a value equal to its fractional percentage in the sample plus the fractional percentage of each less common race being added to the race of interest

With personal health information as the input, the output of our ANN was a fractional number between 0 and 1, with higher values meaning higher cancer risk. To generate the binary cancer status (Yes or No), as shown in Fig 1, it is standard practice to use a cutoff of 0.5, above which our ANN predicts a Yes cancer status. However, the much larger number of non-cancer cases in our data biases the output towards 0. So instead, once the training was complete, we than calculated sensitivity and specificity for the full range of possible cutoff values. Using the training set we selected the cutoff that maximized the sum of sensitivity and specificity. That same value was then applied to the validation data.

We have also applied random forest (RF) and support vector machine (SVM) algorithms to this dataset and compared them to our ANN.

## Results

### Sensitivity, specificity, and AUC of the neural network

For the training set, the sensitivity was 79.8% (95% CI [31], 75.9%-83.6%) and the specificity was 79.9% (95% CI [31], 79.8%-80.1%). The validation set had sensitivity of 75.3% (95% CI [31], 68.9%-81.6%) and specificity of 80.6% (95% CI [31], 80.3%-80.8%).

**Fig 2:**
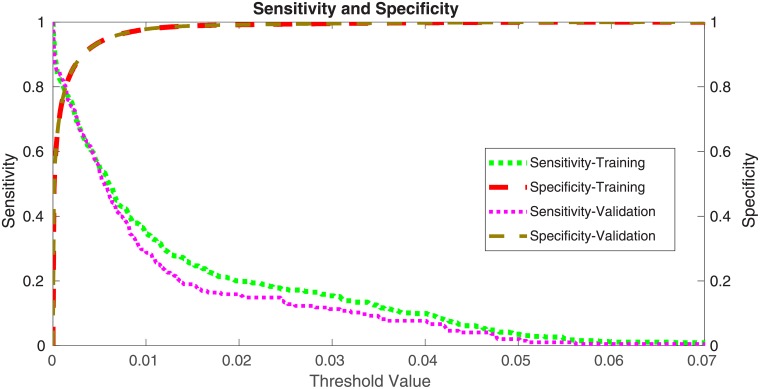
The sensitivity and specificity for the training and validation datasets as functions of the cutoff values.

Since the program computes both sensitivity and specificity for both the training and validations sets, it is important to show how they vary as function of the cutoff value. These results are shown in Fig 2.

**Fig 3:**
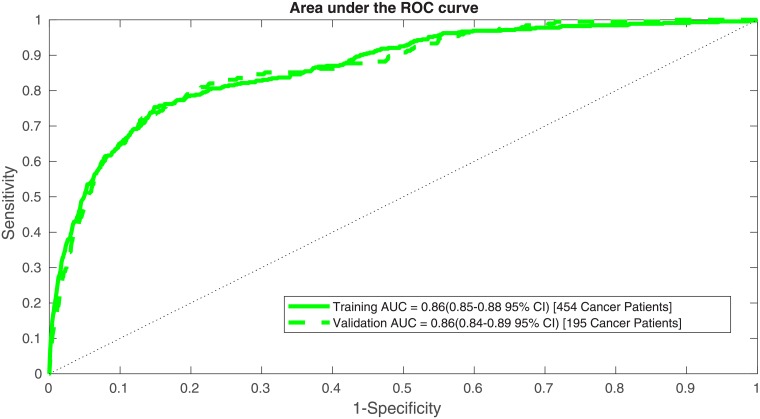
An ROC plot for our ANN’s training and validation datasets.

This information is also conveyed though a conventional receiver operating characteristic (ROC) plot for both the training and validation sets in Fig 3. Our training and validation sets yielded AUC values of 0.86 (95% CI 0.85-0.88) and 0.86 (95% CI 0.84-0.89), respectively.

**Fig 4:**
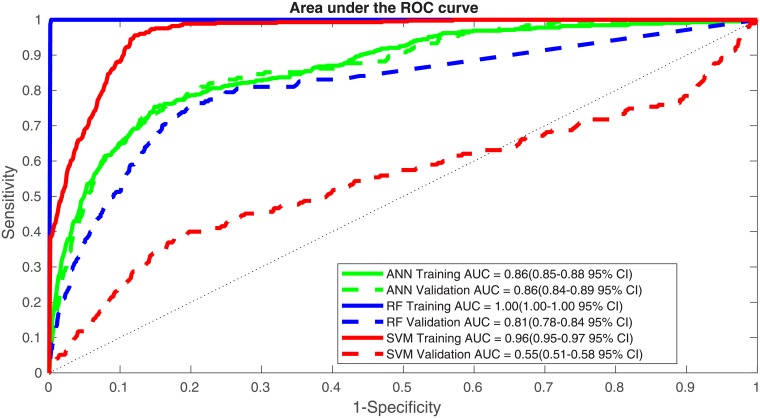
An ROC plot for our ANN’s training and validation datasets as well as the performance of Random Forest and Support Vector Machine.

We also applyed random forest and support vector machine to this same dataset. Both of these methods did better on the training data, with RF having an AUC of 1.00 (95% CI 1.00-1.00) and SVM’s AUC being 0.96 (95% CI 0.95-0.97). However neither of these methods generalized well. The performance of SVM on the validation dataset yielded an AUC 0.55 (95% CI 0.51-0.58). The performance of the RF was better with an AUC of 0.81 (95% CI 0.78-0.84) approaching the performance of the ANN. However the ANN performed the best on the validation dataset and generalized the best (Fig 4).

### A risk stratification tool

While the above results indicate that our model could work well has a diagnostic test, our stated goal was to stratify cancer risk in order to improve screening selection. Accordingly, we present a simple example of how our ANN can be used to do so. When running the ANN instead of applying a cutoff and getting a binary answer, we keep the continuous output from our model and normalize it based on the maximum output from the training and validation sets. This transforms the model output to a percentage equivalent to the cancer risk.

**Fig 5:**
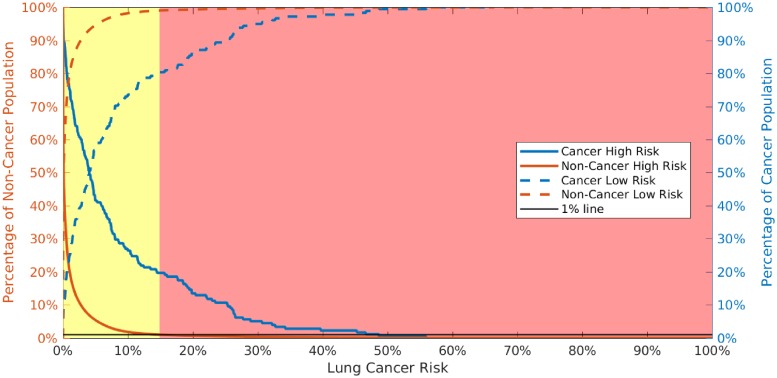
Cumulative distribution function for high risk (solid line) and low risk (dashed line) population without cancer (orange) and population with cancer (blue) populations in the validation dataset. Allowing for a 1% misclassification rate (black line), we can divide individual cancer risk into 3 categories: high (red), medium (yellow), and low (green, too narrow to see on the left of this figure).

As shown in Fig 5, we select two risk boundaries that break the cancer risk into 3 categories: high risk (represented by red), medium risk (yellow), and low risk (green). In this scheme high risk people should be screened immediately, while medium risk people should receive their standard regular screenings (per the ACS recommendations), and low risk people could be screened less frequently. We chose the boundary between medium and high risk so that only 1% of the individuals without cancer would be classified as high risk. Likewise, the boundary between low and medium risk was chosen such that only 1% of the individuals with cancer would be classified as low risk.

We tested the risk stratification scheme with the 2016 NHIS data (27,899 individuals) which was not included in the training or validation of the model. This scheme classifies 18.2% of the population with cancer as high risk who should be screened as soon as possible and 80.0% as medium risk who should be screened according to ACS guidelines. For the population without cancer population, this scheme classifies 12.1% of the population as low risk and 86.8% medium risk (see Table 3). Effectively, our model can be used as a risk stratification tool for clinical decision support.

**Table 3:** NHIS 2016 data risk stratification results by our ANN.

	# People	# Low Risk	% Low Risk	# Medium Risk	% Medium Risk	# High Risk	% High Risk
Cancer	55	1	1.82%	44	80.0%	10	18.2%
Non-Cancer	27,844	3,362	12.1%	24,159	86.8%	323	1.16%

## Discussion

The USPSTF recommends screening with LDCT for early detection of lung cancer in high risk individuals aged 55—80 who have a smoking history of at least 30 pack years and who are current smokers or have quit within the last 15 years [4]. These recommendations are endorsed by the American Cancer Society [6]. The effectiveness of LDCT screening in terms of reducing lung cancer-specific mortality was demonstrated in the National Lung Screening Trial as a 20% reduction in mortality compared to screening with chest radiograph [32]. This mortality benefit has not been reproduced in other studies, but likely reflects the lack of sufficient power in studies smaller than the NLST to do so [33–37]. The NLST study showed a sensitivity of 93.8%, but with a false positive rate of 96.4%. This high false positive rate leads to a significant number of follow-ups, ranging from additional imaging to more invasive procedures such as biopsies. Given the risk of false positive findings, the magnitude of follow-ups generated, and the potential for harm related to possible invasive interventions and additional radiation exposure, the American Cancer Society and other invested organizations recommend screening only for high risk patients at clinics with “access to high-volume, high-quality lung cancer screening and treatment centers”, and only after a discussion relating “the potential benefits, limitations, and harms associated with screening for lung cancer with LDCT.” [4, 6, 35, 38]

Recognizing the limitations posed by LDCT screening, other modalities for lung cancer screening continue to be investigated. These include other imaging modalities [33, 39–44], breath analysis [45], blood tests [46], urine analysis [47], biomarkers [48, 49], and genetic markers [50–55]. While many of these show promise in small studies, few have been tested in large trials. A detailed review of these methods is beyond the scope of this study, however we provide a brief summary of the accuracy, pros, and cons of each method with the corresponding references in Table 4.

**Table 4:** The various screening methods, with their sensitivities and specificities.

Method	Sensitivity	Specificity	Pros and Cons
Our developed ANN	75.3%	80.6%	Noninvasive, Cost-effective, Easy to implement; Less Sensitive than LDCT
Low-Dose CT Scan [56]	93.8%*	73.4%*	Noninvasive, High sensitivity; Expensive, False positives, Radiation exposure
Chest X-ray [56]	73.5%*	91.3%*	Noninvasive; Expensive, False positives, Radiation exposure
Sputum Cytology [48]	16%	99.1%	Noninvasive; Low sensitivity
Automated Sputum Cytometry [48]	40%	91%	Noninvasive, High through-put; Low sensitivity
hnRNP A2/B1 Expression [50]	80.5%	73.5%	High accuracy; Expensive
Promoter Hypermethylation [52]	63%-86%	75%-92%	High accuracy; Expensive
Microarray Gene Spectorometry [45]	80%	84%	High accuracy; Expensive, More invasive
Gas Chromatography-Mass Spectorometry [45]	51%-96.5%	66.7%-100%	Noninvasive, Can be accurate; Expensive, Difficult to perform correctly
Electronic Noses [45]	71.4%-87%	48%-100%	Noninvasive, Can be accurate; Expensive, Difficult to perform correctly
Biomarkers in Blood [46]	41%-77%	80%-93%	Approaching high accuracy; Blood draw and analysis
Buccal Mucosa Analysis [44]	79%	83%	Noninvasive, Quick; Limited testing
Urine Analysis [47]	72%-79%	85%-100%	Noninvasive, High accuracy; Limited testing

* These values are based on three years of screening and follow up on a positive screen. Instead considering each scan or radiograph in isolation the false positive rate goes way up (96.4% and 94.5%) and the positive predictive value drops to 3.8% and 5.7% for Low-Dose CT scans and chest X-rays, respectively [32, 56].

The majority of these methods are proposed as adjuncts to CT screening, to refine the identification of high risk individuals who would most benefit and exclude individuals who would not. Improving identification of appropriate at-risk populations would lower unnecessary radiation exposure and expense as well as relieve the burden of the follow-up examinations, stress, and anxiety from the many false positives of LDCT screening. Our results demonstrate that an artificial neural network can fulfill a similar function, but relaying on data already gathered and a standard computer it is cheap and easy to execute. It is a non-invasive method of predicting lung cancer risk using personal health information (age, BMI, smoking, heart diseases, etc.), and can be distributed into the clinics to support clinical decision-making. The ANN has the advantages of using readily available data with minimal cost and is non-invasive.

Despite the limited amount of data used in our model, it performs very well in identifying patients at risk for lung cancer. With a sensitivity of 75.3% and a specificity of 80.6%, it outperforms all the non-invasive methods listed in Table 4 except the best breath analysis tests. It performs better than chest radiograph and is competitive with many of the other, more intensive methods (e.g., blood test, microarray gene expression, hnRNP A2/B1 Expression). While the ANN does not have as high of a sensitivity as LDCT screening in the USPSTF-selected population, its low false positive rate gives a positive predictive value 10-fold higher than the CT scan (0.395 vs. 0.038).

Furthermore, we showed that the ANN can produce a continuous risk value that can be used for stratification. We presented a simple 3-tiered system that identified almost 20% of the population who could be prioritized for screening. This scheme also classified the majority of those without cancer as medium risk, who we suggest could follow the ACS guidelines for screening. Following the ACS guidelines, based on age and number of pack-years smoked, will likely recommend many of those people not receive LDCT screening. This is equivalent to identifying them as low risk in our scheme. Ideally our model would be able to do this on its own, but our model only uses if someone has ever smoked not the amount they have smoked. We hope to improve our model with more detailed smoking habit information included in the future, but even without it our model still identifies the highest risk population well.

## Conclusion

We have developed and validated a multi-parameterized artificial neural network for lung cancer risk prediction based solely on personal health information readily available in EMR systems. Our results demonstrate that our artificial neural network can offer high specificity and modest sensitivity for identification of lung cancer risk, as compared to other risk predictive modalities currently employed. This approach is cheap, non-invasive, and easy to implement. While our neural network could be potentially used as a clinical tool for lung cancer risk stratification, further improvement with more risk factors included and more clinical testing would be needed.
